# Antibacterial Activity of Essential Oils Combinations based on *Thymus broussonnetii*, and Their Synergism with some Antibiotics

**DOI:** 10.1007/s00284-023-03510-x

**Published:** 2023-11-01

**Authors:** Oumaima Amassmoud, Imane Abbad, Marcello Iriti, Lahcen Hassani, Noureddine Mezrioui, Abdelaziz Abbad

**Affiliations:** 1 Laboratory of Microbial Biotechnologies, Agrosciences and Environment, Faculty of Sciences Semlalia, (Labeled Research Unit‑CNRST N°4), Cadi Ayyad University, Marrakech, Morocco; 2 Faculty of Sciences Semlalia, University Cadi Ayyad, Marrakech, Morocco; 3 Department of Biomedical, Surgical and Dental Sciences, Università Degli Studi Di Milano, 20142 Milan, Italy; 4 National Interuniversity Consortium of Materials Science and Technology, Via G. Giusti 9, 50121 Florence, Italy

## Abstract

The present study aimed to evaluate the antibacterial activity of the essential oil (EO) of Moroccan endemic *Thymus broussonnetii* alone, and in combination with EOs obtained from selected medicinal plants, namely *Myrtus communis*, *Artemisia herba alba*, *Thymus pallidus*, *Thymus satureioides*, *Teucrium polium,* and *Rosmarinus officinalis*. The synergistic interactions between the most effective combinations based on *T. broussonnetii* EO with two conventional antibiotics (streptomycin and ciprofloxacin) were also investigated. *T. broussonnetii* EO possessed a higher inhibitory activity against tested pathogenic bacteria with inhibition zone diameter (IZ) ranging from 21.61 ± 0.03 to 40.09 ± 0.02 mm, and MIC values between 0.140 mg/mL and 0.280 mg/mL. *M. communis*, *A. herba alba*, *T. pallidus*, *T. satureioides*, *T. polium,* and *R. officinalis* EOs showed moderate to weak antibacterial activity. Among tested EO mixtures, the highest synergistic antibacterial effect was recorded with the EO combination of *T. broussonnetii* and *T. pallidus* against *S. aureus*, *E. coli,* and *S. enterica* (FICI = 0.258). This EO combination was also the most effective mixture to synergistically enhance the antibacterial activity of the two antibiotics with up to a 128-fold increase, particularly against the gram-negative *E. coli*. These findings suggest that *T. broussonnetii* EO may be an interesting source of natural antimicrobials, for use in combination therapies with other plant EOs, and with conventional antimicrobial drugs to tackle the emergence of multidrug-resistant bacteria.

## Introduction

Bacterial resistance to conventional antimicrobial agents (AMR) has been described as one of the world’s major healthcare concerns, as it is associated with increased morbidity, mortality, and economic loss [1]. AMR is estimated to be result in around 33,000 deaths in the EU and 700,000 deaths globally per year, with annual economic costs of 1.5 billion Euros in the EU [2]. It has been estimated that globally AMR might cause more deaths than cancer by 2050 with a projected cost to be around 100 trillion dollars [1]. This healthcare problem requires the development of novel effective antimicrobial materials tools to limit the usage of current antibiotics, and to relieve the burden of infection [3]. With no new drugs in the pipeline, essential oils (EOs) have emerged as alternative antimicrobial products due to their strong and wide-spectrum activity against microorganisms, in addition to their ecofriendly and human safety status [4, 5]. Many medicinal plants with their secondary metabolites (including EOs) form the basis for commercial pharmaceutical drugs and herbal remedies. However, the doses of these EOs required to achieve an effective antimicrobial activity may exceed consumer’s preference [4]. To counteract this problem in the context of AMR, combination of EOs with traditional antibiotics to enhance their effectiveness and consequently to decrease their efficacious doses has been investigated [6–8]. Many recent studies report that the synergistic combination of EOs and conventional antimicrobials has proved to be an effective solution and a practical alternative to develop drugs with increased efficacy and low toxicity [5, 7, 9]. Their inherent ability to suppress development of resistance has been attributed to some plant EOs being able to sensitize bacteria to the action of antibiotics [7]. In addition, EOs with their complex mixtures containing different compounds with variable chemical structures, can act through different mechanisms and against target sites compared to antibiotics which commonly target relatively few sites [7]. Different mechanisms of synergy exhibited by EO-antibiotic combinations have been suggested, including the inhibition of efflux pumps implicated in exporting antimicrobials outside the bacterial cells, the inhibition of the production or activity of enzymes (e.g., beta lactamases, lipases, coagulases, and/or amino acid decarboxylases), the permeabilization and disruption of bacterial membranes by inhibiting ATPase activity, the alteration of the cell wall composition, the inhibition of membrane-located metabolic events leading to inhibition of respiration, leakage of cell ions, and reduction of proton motive force, among others [5, 10–13].

*Thymus* species are of special interest with respect to the investigation of their natural antimicrobial agents and the possible synergistic effect of their plant volatile extracts with antibiotics, mainly due to their richness in EOs, and their strong antimicrobial activity [14, 15]. Morocco is rich in *Thymus* species with 22 species, of which 15 are endemic [16]. Among the endemic species, *T. broussonnetii* Boiss., known locally as “Zaater Essouiri,” is a spontaneous, perennial aromatic shrub extensively used in Moroccan folk medicine, and as an excellent food preservative [17]. Previous investigations have shown interesting antimicrobial activity and a high level of synergism by *T. broussonnetii* EO with some antibiotics [13, 18]. To our knowledge, no information is available on the antibacterial synergistic potential of *T. broussonnetii* EO with some other medicinal plant EOs, or on the combinational effect of EO mixtures based on this valuable *Thymus* species with conventional antimicrobials. As far as we know, this is the first study to improve the antibiotic efficacy by some effective EO mixtures based on *T. broussonnetii* EO. Thus, the present work aimed to investigate the combined antimicrobial potential of *T. broussonnetii* EO with some selected medicinal plant EOs, namely *Myrtus communis*, *Artemisia herba alba*, *Thymus pallidus*, *Thymus satureioides*, *Teucrium polium,* and *Rosmarinus officinalis*. We also investigated the antibacterial effect of the most effective EO mixtures based on *T. broussonnetii* EO in combination with two commonly used antibiotics (streptomycin and ciprofloxacin).

## Materials and Methods

### Plant material and EO Extraction

Aerial parts of *M. communis*, *A. herba alba*, *T. pallidus*, *T. satureioides*, *T. polium*, *R. officinalis,* and *T. broussonnetii* were harvested from different rural locations in Morocco (Table 1). The medicinal plants used in the present work were selected based on their therapeutic usages and antimicrobial properties. In fact, *Thymus* species, in particular, *T. broussonnetii*, *T. pallidus,* and *T. satureioides* aerial parts are well known in Moroccan folk medicine for their high antimicrobial and antiseptic properties and for the treatment of many infections such as gastrointestinal, respiratory, genital, and skin diseases. Because of their antiseptic activities, the aerial part of *R. officinalis*, *M. communis,* and *A. herba alba* are also widely used as herbal remedies for the treatment of chronic wounds, eye, and gastrointestinal infections, respectively [17]. The identification of plant species was undertaken by one of the authors (A. Abbad), and voucher specimens have been deposited at the Laboratory of Microbial Biotechnologies, Agrosciences and Environment of the Faculty of Science Semlalia, University Cadi Ayyad, Marrakech. The plant samples were dried at ambient temperature (≈25 °C) for EO isolation. Each dried plant material was submitted to three successive steam distillations (3 × 200 g) for about 3 h using a Clevenger-type apparatus, and the resulting oils were dried with anhydrous sodium sulfate (Na_2_SO_4_), weighed, and kept at 4 °C for further analysis. The EO yields were expressed in % (v/w) of the dry material using the following equation: % Yield of the EO = (Volume of the extracted EO (mL) ∕ Weight of the dried plant material (*g*)) × 100.

**Table 1:** Collection site and geographical coordinates of the studied Moroccan aromatic plants and their EO yields

Species	Species code	Local name	Collection site	Harvesting time	Voucher specimen	Latitude/Longitude	Altitude (m)	Oil Yield (%) (v/w)
*Myrtus communis*	MC	Rihane	Ouezzane	May 2022	MC-10	34°47’ N/05°34’ W	1450	0.35 ± 0.18
*Artemisia herba alba*	AHA	Chih	Ijoukak	May 2021	AHA-43	30°59′ N/08°09′ W	1280	0.30 ± 0.29
*Thymus pallidus*	TP	Zaitra	Ait Lkak	June 2021	TP-34	31°17’ N/07°50’ W	1750	1.49 ± 0.22
*Thymus satureioides*	TS	Zaitra	Ourika	May 2021	TS-32	31°14’ N/07°41’ W	1460	2.17 ± 0.03
*Teucrium pollium*	TPo	Jaïdiya	Ounagha	Avril 2022	TPo-21	31°33’ N/09°33’ W	280	0.20 ± 0.21
*Rosmarinus officinalis*	RO	Azir	Rich	Sept. 2021	RO-13	32°16’ N/04°36’ W	1540	2.22 ± 0.12
*Thymus broussonetii*	TB	Zaater Essouiri	Essaouira	Avril 2022	TB-45	31°29’ N/09°33’ W	196	2.80 ± 0.45

### GC/FID and GC/MS Analysis

The qualitative and quantitative identifications of the EO constituents were conducted using gas chromatography with Flame Ionization Detection (GC/FID) and gas chromatography coupled to mass spectrometry (GC/MS) analysis. The GC/FID was performed using a Shimadzu GC-2010 instruments equipped with an SLB-5MS Supelco (Milan, Italy), 30 m × 0.25 mm i.d. × 0.25 μm d, coated with 5% diphenyl-95% polydimethylsiloxane. Helium (flow rate 1 mL/mn) was used as a carrier gas. The initial oven temperature was 50 °C, the final temperature was 270 °C, and the temperature gradient was 4 °C/min. The injector and detector temperature were 280 °C and the injected volume of EO was 2.0 μL using the split mode. The GC/MS analysis was carried out using a gas chromatograph equipped with a TG-5MS column (length: 30 m; internal diameter: 0.25 mm, thickness film: 0.25 µm), and coupled to a mass selective detector ISQ (Single Quadrupole Mass spectrometer). The oven temperature was programmed to rise from 60 to 246 °C at 3 °C/min. Transfer, source, and quadrupole temperatures were 280 °C, 230 °C, and 150 °C, respectively, operating at 70 eV ionization energy and scanning the m/z range 41–450. EOs samples (20 μL) were diluted with hexane (2mL). 1 μL was injected at 260 °C in split mode (1/50). Identification of the individual components was based on the comparison of their mass spectra with NIST MS Search database and Adams terpene library, and comparison of their retention indices (RI) on a TG-5MS. The RI was calculated relative to the retention times of a series of n-alkanes (C5-C30), using the Van den Dool and Kratz equation [19], with linear interpolation with those of authentic compounds or literature data. For semi-quantification purposes, the normalized peak area of each compound was used without any correction factors to establish abundances. RI and peak area percentages were calculated as mean values of three injections.

### Determination of Antibacterial Activity

#### Chemicals and Culture Media

Antibiotics selected for this study (streptomycin and ciprofloxacin) and chemicals (DMSO) were purchased from Sigma-Aldrich, while culture media, MHA, and MHB were obtained from Biokar.

#### Antibacterial Screening

The individual EOs and their binary combinations were first screened for their antibacterial activity against six bacterial pathogenic strains provided by the American type culture collection through the *QualiUp* Groupe (Agadir, Morocco), known for their low susceptibility to most conventional antimicrobials [13]: *Staphylococcus aureus* (ATCC 6538), *Bacillus subtilis* (ATCC 6633), *Escherichia coli* (ATCC 25922), *Enterococcus faecalis* (ATCC19433), *Listeria monocytogenes* (ATCC13932), and *Salmonella enterica* (ATCC 14028).

The screening of antibacterial activity of each EO was evaluated using the agar disk diffusion method following the CLSI instructions with some modifications [20]. For this, sterile 6 mm diameter disks impregnated with 10 µL of each EO were applied to the surface of MHA plates previously seeded with 0.1 mL of bacterial suspensions at 10^8^ CFU/mL, equivalent to 0.5 McFarland. All plates were kept at 4°C for 2 h in order to allow EOs diffusion, and then incubated at 37 °C for 18–24 h. After incubation, IZ were measured in mm (including disk diameter). Streptomycin (10 μg/disk) and ciprofloxacin (5 μg/disk) were used as positive controls. All assays were carried out in triplicate.

#### Determination of MIC and MBC

Quantitative analyses of EO antibacterial activity were evaluated using the broth microdilution method according to the CLSI guidelines M07-A10 [21], with slight modifications. Briefly, 100 µL of twofold serial dilutions of each EO (previously prepared in MHB using 1% DMSO) were added to microwells containing 100 µL of bacterial suspensions at 10^8^ CFU/mL. DMSO was used as negative control. The microplates were incubated for 24 h at 37 °C, and MICs (mg/mL) were determined as the lowest concentration of the oils contributing to inhibition of macroscopic bacterial growth. To determine the MBC (mg/mL), 0.1 mL of all clear wells which did not show any visible growth during MIC assays were spread on MHA plates, and incubated at 37 °C for 24 h. The MBC was defined as the lowest concentration of the EO required to kill 99.99% of the incubated bacteria [20]. All assays were conducted in triplicate. Streptomycin and ciprofloxacin were used as standard antibacterial drugs. A negative control was prepared using a concentration of 1% DMSO.

#### Checkerboard Method

The Checkerboard method is an in vitro technique used to determine the activity of combination of antimicrobial substances. In this study, the binary antibacterial interactions between *T. broussonnetii* EO at MIC/4 as previously determined [14], and other selected medicinal plant EOs against some pathogenic bacteria were investigated as described by Kwiatkowski et al. [22]. 50 µL of *T. broussonnetii* EO at MIC/4 was added to each well, containing 50 µL of eight serial twofold dilutions (from MIC to MIC/128) of each other plant EO, in such a way that each well contained a unique combination of the two different EOs. Then, 100 µL of bacterial suspensions at 10^8^ CFU/mL, equivalent to 0.5 McFarland, were added to each well and incubated at 37 °C for 24h. The FICI used for the determination of the nature of the interaction between two EOs was calculated following the formula described by Skroza et al. [23]. The interactions between the most effective EO binary combinations based on *T. broussonnetii* EO, and the antibiotics were carried out using the same methodology. Briefly, 50 µL of each effective EO combination at MIC/4 was added to each well, containing 50 µL of eight serial twofold dilutions (from MIC to MIC/128) of antibiotics in such a way that each well contained a unique combination of a binary EO mixture (50 µL) and an antibiotic (50 µL). Then, 100 µL of bacterial suspensions at 10^8^ CFU/mL, equivalent to 0.5 McFarland, was added to each well, and incubated at 37 °C for 24h.

FICI was calculated as follows:

$${\text{FICI}} = {\text{ FIC }}\left( {{\text{EO}}_{{1}} } \right) \, + {\text{ FIC }}\left( {{\text{EO}}_{{2}} } \right)$$

With FIC (EO_1_) = MIC of EO_1_ in combination with EO_2_/MIC of EO_1_ alone, and FIC (EO_2_) = MIC of EO_2_ in combination with EO_1_/MIC of EO_2_ alone.

The results were interpreted as following: synergism (FICI ≤ 0.5), addition (0.5 < FICI ≤ 1.0), neutral (1 < FICI ≤ 4.0), or antagonism (FICI > 4.0).

The MIC gain of the antibiotics was calculated as the antibiotic MIC alone divided by the MIC of antibiotic combined with EO.

The FICI values of the effective EO combinations and antibiotics against the tested bacterial strains were determined according to the same formula.

#### Statistical Analysis

Data were expressed as the mean ± Standard deviation, group means were compared by one-way ANOVA and Tukey test to identify significance (p < 0.05) among groups using SPSS 21.0 statistical software.

## Results

### Yield and Chemical Composition of EOs

The yield of EOs isolated by steam distillation from aerial parts of the studied plants varied from 0.2% ± 0.21 to 2.80% ± 0.45 (Table 1). The EO chemical compositions analyzed by GC-SM led to the identification of 56 compounds accounting for between 86.69 and 99.16% of the total oils. Monoterpene hydrocarbons constituted the principal sub-class in *T. satureioides* EO, while oxygenated monoterpenes were dominant in *M. communis*, *A. herba alba*, *T. pallidus*, *R. officinalis,* and *T. broussonnetii* EOs. In *T. polium EO*, the dominant chemical family was sesquiterpene hydrocarbons.

With respect to particular chemical constituents, *M. communis* and *R. officinalis* were dominated by 1,8-cineole at 42.00% and 45.79%, respectively. In addition to this oxygenated monoterpene, *M. communis* EO was characterized by the presence of α-pinene (19.73%) and myrtenyl acetate (23.63%) as other major constituents, while camphor (21.86%) was the other main component of *R. officinalis* EO (Table 2). EOs obtained from *T. pallidus*, *T. satureioides,* and *T. broussonnetii* were dominated, respectively, by thymol (25.12%)/p-cymene (18.74%)/γ-terpinene (15.99%), borneol (25.10%)/camphene (23.20%)/p-cymene (13.70%)/α-pinene (11.50%), and carvacrol (44.39%)/thymol (21.47%)/γ-terpinene (9.09%). The most abundant compounds in *A. herba alba* were β-thujone (37.83%), α-thujone (23.70%), and camphor (12.70%), while the main constituents of *T. polium* EO were germacrene (29.4%), α-pinene (18.05%), and bicyclogermacrene (14.5%).

**Table 2:** Chemical composition of the EOs isolated from the aerial parts of studied Moroccan aromatic plants

RT	RI	Compounds^a^	Content (%)						
			Mc^b^	Aha	Tp	Ts	Tpo	Ro	Tb
5.81	925	Tricyclene	–^c^	–	–	1.35	–	–	–
5.91	928	β–Thujene	–	0.17	0.14	0.20	–	–	1.27
6.11	931	α–Pinene	19.73	0.19	0.21	11.50	18.05	8,68	2.20
6.41	950	Camphene	0.17	1.83	0.32	23.20	0.9	2,56	0.27
6.69	980	4–Hydroxyhexenal	–	0.19	–	–	–	–	–
7.77	986	Myrcene	0.69	–	0.94	–	6.92	0.65	1.60
7.80	999	α–Thujene	–	0.58	–	–	0.6	0.13	0.20
7.99	1002	Boldione	–	0.22	–	0.24	–	–	–
8.22	1007	α–Phellandrene	0.22	–	–	–	–	–	0.20
8.29	1018	α–Terpinene	0.32	–	–	0.73	–	–	–
8.39	1022	1,3,8–p–Menthatriene	–	0.63	–	–	–	–	–
8.64	1030	δ–3–Carene	0.12	–	–	–	–	–	1.47
8.92	1033	p–Cymene	0.23	–	18.74	13.70	1.2	0.82	6.39
9.07	1035	Limonene	–	–	0.19	1.70	8.59	–	0.66
9.18	1042	1,8–Cineole	42.00	3.03	–	–	–	45.79	–
10.17	1050	γ–Terpinene	0.34	–	15.99	1.56	–	0.59	9.09
10.20	1155	Menthone	0.35	–	–	–	–	–	–
10.29	1069	β–Thujone	–	37.83	0.66	–	–	–	–
10.48	1086	Sabinene hydrate	–	–	–	–	–	–	0.23
10.59	1087	Butyric acid	0.23	0.27	–	–	–	–	–
11.32	1089	α–Terpinolene	–	–	–	–	–	0.15	–
11.75	1090	Linalool	2.95	–	6.06	3.41	0.52	1.00	0.17
12.41	1113	α–Thujone	–	23.70	–	–	–		–
12.59	1120	Isothujol	–	0.24	–	–	–	–	–
12.73	1127	Chrysantenone	–	0.70	–	–	–	–	–
13.51	1140	Camphor	–	12.70	–	0.50	–	21.86	–
14.28	1160	Pinocarvone	–	0.65	–	–	0.9	–	–
14.40	1169	Borneol	–	1.35	8.06	25.1	1.63	2.26	1.22
14.94	1173	4–Terpineol	0.35	0.25	0.46	0.72	–	1.37	0.21
15.71	1187	Myrtenal	–	0.22	–	1.62	0.85	–	–
16.30	1192	α–Terpineol	2.95	–	–	3.41	–	–	–
17.02	1199	Mythenal					1.05		
18.43	1248	Chrysanthenyl acetate	–	0.29	–	–	–	–	–
19.46	1283	Bornyl acetate	0.50	0.53	–	3.50	0.52	–	–
20.09	1288	Thymol	0.88	0.20	25.12	0.41	2.12	3.22	21.47
20.17	1323	Myrtenyl acetate	23.63	–	–	–	–	–	–
20.30	1327	Carvacrol	–	–	–	4.50	–	–	44.39
20.45	1350	α–Terpinyl acetate	–	–	–	–	–	–	–
22.50	1381	α–Copaene	–	–	–	0.69	–	–	–
22.60	1384	Geranyl acetate	2.73	–	–	–	0.28	–	–
24.50	1398	β–Bourbonene	–	–	–	–	1.2	–	–
25.02	1425	Caryophyllene	–	–	6.05	–	–	5.88	2.59
26.38	1453	Humulene	0.20	–	–	–	0.92	0.52	–
26.79	1469	Spatulenol	–	–	–	–	2.63	–	0.67
27.47	1572	Germacrene	–	–	–	0.17	29.4	–	0.26
27.80	1540	α–Ocimene	–	–	–	–	–	–	0.12
28.80	1555	γ–Cadinene	–	–	1.94	–	–	–	–
29.67	1560	Aromadendrene	–	–	–	–	–	–	0.16
31.43	1580	Caryophyllene–oxide	–	–	3.10	–	–	–	–
31.50	1590	Bicyclogermacrene	–	–	–	–	14.5	–	2.57
31.76	1599	Trans–Arbusculone	–	0.92	–	–	–	–	–
32.65	1602	Epicubenol	–	–	0.69	–	–	–	–
33.60	1620	α–Cadinol	–	–	2.70	–	0.92	–	–
47.90	1720	Prasterone acetate	–	–	0.44	–	–	–	–
49.45	1735	Copalic acid	–	–	3.81	–	–	–	–
59.5	2016	Eugenol methyl ether	0.57	–	–	–	–	–	–
Total identified compounds %	99.16	86.69	95.62	98.21	93.7	95.48	97.41		
Monoterpene hydrocarbons	38.65	2.77	36.53	53.94	36.26	13.58	22.81		
Oxygenated monoterpenes	72.54	79.06	40.36	33.55	4.55	75.5	67.69		
Sesquiterpene hydrocarbons	0.2	0	7.99	0.86	44.82	6.4	5.58		
Oxygenated sesquiterpenes	0.57	0	5.8	0	0.92	0	0		
Other	1.08	4.86	5.6	5.36	7.15	0	0.67		

RT, retention times; RI, Retention indices determined using the homologous series of n-alkanes

^a^Compounds listed in order of elution

^b^Codes of species as listed in Table 1

^c^not detected

### Antibacterial Activity of Individual EOs

The in vitro antibacterial activity of the tested EOs against *S. aureus, B. subtilis, E. coli, E. faecalis, L. monocytogenes,* and *S. enterica* was determined using disk diffusion and broth microdilution assays. All tested EOs exhibited varying levels of activity against these bacterial species. In general, the EOs obtained from *Thymus* species showed excellent antibacterial activity, with IZ and MIC values ranging from 13.99 ± 0.15 to 40.09 ± 0.13 mm and 0.140 to 1.125 mg/mL, respectively (Tables 3 and 4). *T. broussonnetii* EO displayed the highest antibacterial efficacy (IZ = 21.61 ± 0.03–40.09 ± 0.02 mm and MIC = 0.140–0.280 mg/mL), which was surprisingly, more active than the tested antibiotics against some bacteria strains (Tables 3 and 4). *M. communis* and *R. officinalis* EOs exhibited moderate inhibitory effects (IZ = 7.77 ± 0.19–14.38 ± 0.13 mm and MIC = 4.5–144 mg/mL for *M. communis*, and IZ = 6.47 ± 0.21–14.00 ± 0.06 and MIC = 1.125 mg/mL–> 144 mg/mL for *R. officinalis*). The weakest antibacterial activity against the tested bacteria was observed with *A. herba alba* and *T. polium* EOs (Tables 3 & 4). The antibacterial results also showed that the all EOs were more effective against the Gram-positive bacteria than the Gram-negative ones. Among the Gram-positive bacteria screened, *S. aureus* was the most sensitive to all tested EOs.

**Table 3:** Inhibition zone of tested EOs and selected antibiotics against six bacterial strains

EOs/antibiotics	Bacterial strains					
	*S. aureus*	*B. subtilis*	*E. coli*	*E. faecalis*	*L. monocytogenes*	*S. enterica*
	IZ (mm)					
*Myrtus communis*	14.38^e^ ± 0.13	9.00^f^ ± 0.08	7.96^f^ ± 0.09	8.90^f^ ± 0.20	10.87^f^ ± 0.05	7.77^e^ ± 0.19
*Rosmarinus officinalis*	14.00^f^ ± 0.06	8.12^g^ ± 0.09	7.15^g^ ± 0.12	7.00^h^ ± 0.20	9.20^g^ ± 0.01	6.47^f^ ± 0.21
*Thymus pallidus*	30.76^b^ ± 0.08	22.01^c^ ± 0.18	18.89^c^ ± 0.58	19.56^b^ ± 0.04	36.26^b^ ± 0.03	20.58^c^ ± 0.23
*Thymus satureioides*	29.48^c^ ± 0.06	14.01^e^ ± 0.13	13.99^e^ ± 0.15	15.46^d^ ± 0.05	15.07^e^ ± 0.04	14.41^e^ ± 0.07
*Teucrium polium*	10.87^g^ ± 0.40	6.00^h^ ± 0.00	6.00^h^ ± 0.00	6.00^i^ ± 0.00	9.16^g^ ± 0.03	6.06^f^ ± 0.04
*Artemisia herba alba*	7.44^h^ ± 0.06	6.00^h^ ± 0.00	7.35^g^ ± 0.12	8.24^g^ ± 0.05	8.38^h^ ± 0.14	7.40^e^ ± 0.14
*Thymus broussonnetii*	40.09^a^ ± 0.02	25.64^b^ ± 0.01	25.66^b^ ± 0.38	21.61^a^ ± 0.03	40.07^a^ ± 0.13	27.16^a^ ± 0.63
Streptomycin	17.85^d^ ± 0.03	15.23^d^ ± 0.48	16.43^d^ ± 0.01	13.08^e^ ± 0.03	19.80^c^ ± 0.22	17.08^d^ ± 0.01
Ciprofloxacin	29.29^c^ ± 0.05	37.64^a^ ± 0.07	33.48^a^ ± 0.02	16.45^c^ ± 0.03	19.14^d^ ± 0.03	26.10^b^ ± 0.04

IZ, diameter of inhibition zone including disk diameter of 6 mm at a concentration of 10 μL of oil/disk, and a concentration of 10 μg/disk of streptomycin and 5 μg/disk of ciprofloxacin. Means with different letters within a column are significantly different (p < 0.05)

**Table 4:** Minimal inhibition concentration (MIC) and minimal bactericidal concentration (MBC) of tested EOs and antibiotics against six bacterial strains

EOs/antibiotics	Bacterial strains											
	*S. aureus*		*B. subtilis*		*E. coli*		*E. faecalis*		*L. monocytogenes*		*S. enterica*	
	MIC	MBC	MIC	MBC	MIC	MBC	MIC	MBC	MIC	MBC	MIC	MBC
*Myrtus communis*	4.5	4.5	36	72	72	144	72	144	2.5	2.5	144	144
*Rosmarinus officinalis*	1.125	4.5	72	144	144	> 144	72	144	4.5	9	> 144	> 144
*Thymus pallidus*	0.140	0.140	0.281	0.281	0.281	0.281	0.563	0.563	0.140	0.563	1.125	1.125
*Thymus satureioides*	0.563	1.125	0.563	0.563	0.563	0.563	0.563	1.125	0.563	1.125	2.25	2.25
*Teucrium polium*	2.25	4.5	72	144	144	> 144	144	> 144	36	72	72	144
*Artemisia herba alba*	72	72	72	144	144	144	72	144	> 144	> 144	72	144
*Thymus broussonnetii*	0.140	0.281	0.140	0.281	0.281	0.281	0.140	0.281	0.281	0.281	0.281	0.281
Streptomycin	9	18	9	9	72	72	144	144	9	9	72	72
Ciprofloxacin	72	72	4.5	9	4.5	4.5	288	288	288	288	72	72

MIC and MBC are in mg/mL for EOs and in μg/mL for antibiotics

### Antibacterial Activities of Different EO Combinations

Synergistic interactions between *T. broussonnetii* at MIC/4 and selected plant EOs were determined using the checkerboard method. The binary combinations of these EOs were tested against the six bacterial species assayed for sensitivity test to EOs (viz. *S. aureus, B. subtilis, E. coli, E. faecalis, L. monocytogenes,* and *S. enterica*). The results showed that from the 34 tested combinations, 9 (26.47%) displayed synergism and 15 (44,12%) had neutral interactions (Table 5). The greatest synergistic effect was observed with the EO combination of *T. broussonnetii* and *T. pallidus* against *S. aureus*, *E. coli,* and *S. enterica*, with FICI = 0.258 (Table 5). The synergistic effect of *T. broussonnetii* in combination with the other plant EOs varied in relation to the bacterial species tested. Against *S. aureus*, except for *A. herba alba* EO, all other EOs showed synergism in combination with *T. broussonnetii* EO. In addition, the combination of *T. broussonnetii* and *R. officinalis* or *M. communis* EOs showed synergism against *E. faecalis* and *L. monocytogenes*, respectively (Table 5).

**Table 5:** Fractional inhibitory concentration index (FICI) of most effective binary combinations of *T. broussonnetii* EO (at MIC/4) with other plant EOs

EO combinations	Bacterial strains																	
	*S. aureus*			*B. subtilis*			*E. coli*			*E. faecalis*			*L. monocytogenes*			*S. enterica*		
	MIC_o_	MIC_c1_	FICI	MIC_o_	MIC_c1_	FICI	MIC_o_	MIC_c1_	FICI	MIC_o_	MIC_c1_	FICI	MIC_o_	MIC_c1_	FICI	MIC_o_	MIC_c1_	FICI
Tb/MC^c^	4.5	0.563	**0.375**^**a**^	36	> 36	–	72	> 72	–	72	72	1.250^b^	2.5	1.125	**0.500**^**a**^	> 144	–	–
Tb/Ro	1.125	0.070	**0.312**^**a**^	72	72	1.250^b^	144	> 144	–	18	4.5	**0.500**^**a**^	4.5	4.5	1.250^b^	> 144	–	–
Tb/Ts	0.563	0.070	**0.375**^**a**^	0.563	0.563	1.250^b^	0.563	0.563	1.250^b^	0.563	0.563	1.250^b^	0.563	> 0.563	–	2.25	2.25	1.250^b^
Tb/Tp	0.140	0.001	**0.258**^**a**^	0.281	0.281	1.250^b^	0.281	0.002	**0.258**^**a**^	0.563	0.563	1.250^b^	0.140	> 0.140	–	1.125	0.0087	**0.258**^**a**^
Tb/Tpo	2.25	0.281	**0.375**^**a**^	72	> 72	–	144	> 144	–	144	144	1.250^b^	36	36	1.250^b^	72	72	1.250^b^
Tb/Aha	72	72	1.250^b^	72	> 72	–	144	144	1.250^b^	72	72	1.250^b^	144	> 144	–	72	> 72	–

MIC_o_, MIC of the individual EOs in mg/mL; MIC_c1_, MIC of EOs in combination in mg/mL; FICI, Fractional inhibitory concentration index; – not-tested

Bold values repersent synergism between EO combinations

^a^synergism

^b^indifference

^c^Codes of species as listed in Table 1

## Synergistic Interaction Between Effective EOs Combinations and Antibiotics

The synergistic antibacterial properties of effective EO mixtures based on *T. broussonnetii* EO (at MIC/4) with two conventional antibiotics (streptomycin and ciprofloxacin) against *S. aureus*, *E. coli*, *E. faecalis*, L. *monocytogenes,* and *S. enterica* were investigated using the checkerboard method. The results showed varying degrees of synergistic interaction between the antibiotics and the EO mixtures depending on bacterial strains and combination type (Tables 6 & 7). The greatest synergistic antibacterial effects were obtained with the combination of *T. broussonnetii* and *T. pallidus* EOs with the two tested antibiotics streptomycin and ciprofloxacin. The association of this EO mixture with streptomycin achieved gains of 8-, 64-, and 128-fold for *S. aureus*, *S. enterica,* and *E. coli*, respectively, while with ciprofloxacin, the gains reached 64-and 128-fold for *S. enterica* and *E. coli*, respectively (Tables 6 and 7). Interestingly, the *T. broussonnetii* and *M. communis* EO mixture showed high synergy with the two antibiotics with a gain of 128-fold against *L. monocytogenes* (Tables 6 and 7). Other synergisms between the mixtures of *T. broussonnetii* with *R. officinalis*, *T. polium,* or *T. satureioides* Eos, and the antibiotics were also observed, with a gain of fourfold against *S. aureus*.

**Table 6:** Fractional inhibitory concentration index (FICI) of streptomycin and most effective EO combinations of *T. broussonnetii* with some selected medicinal plants

Essential oil/antibiotics	Bacterial strains																			
	*S. aureus*				*E. coli*				*E. faecalis*				*L. monocytogenes*				*S. enterica*			
	MIC_c1_/MIC_o_	MIC_c2_	FICI	Gain	MIC_c1_/MIC_o_	MIC_c2_	FICI	Gain	MIC_c1_/MIC_o_	MIC_c2_	FICI	Gain	MIC_c1_/MIC_o_	MIC_c2_	FICI	Gain	MIC_c1_/MIC_o_	MIC_c2_	FICI	Gain
Tb/MCc	0.563		1.25^b^		–	–	–	–	–	–	–	–	1.125		0.26^a^		–	–	–	–
Streptomycin	9	9		**1**	–	–	–	–	–	–	–	–	9	0.070	–	**128**	–	–	–	–
Tb/RO	0.0702		0.5^a^		–	–	–	–	4.5		0.5^a^		–	–	–	–	–	–	–	–
Streptomycin	9	2.25		**4**	–	–	–	–	144	**36**		**4**	–	–	–	–	–	–	–	–
Tb/Ts	0.0702		0.5^a^		–	–	–	–	–	–	–	–	–	–	–	–	–	–	–	–
Streptomycin	9	2.25		**4**	–	–	–	–	–	–	–	–	–	–	–	–	–	–	–	–
Tb/Tp	0.0011				0.0022				–	–	–	–	–	–	–	–	0.0087			
Streptomycin	9	1.13	0.38^a^	**8**	72	0.563	0.26a	**128**	–	–	–	–	–	–	–	–	72	1.13	0.27^a^	**64**
Tb/Tpo	0.281				–	–	–	–	–	–	–	–	–	–	–	–	–	–	–	–
Streptomycin	9	2.25	0.5^a^	**4**	–	–	–	–	–	–	–	–	–	–	–	–	–	–	–	–

MIC_o_, MIC of the individual EOs in mg/mL; MIC_c1_, MIC of EOs in combination in mg/mL; MIC_c2_, MIC of EOs combinations in association with antibiotics in µg/mL; FICI, Fractional inhibitory concentration index; – not-tested; ^a^synergism; ^b^indifference; ^c^Codes of species as listed in Table 1

**Table 7:** Fractional inhibitory concentration index (FICI) of ciprofloxacin and most effective EO associations of *T. broussonnetii* with some selected medicinal plants

EO combinations/ antibiotics	Bacterial strains																			
	*S. aureus*				*E. coli*				*E. faecalis*				*L. monocytogenes*				*S. enterica*			
	MIC_c1_/MIC_o_	MIC_c2_	FICI	Gain	MIC_c1_/MIC_o_	MIC_c2_	FICI	Gain	MIC_c1_/MIC_o_	MIC_c2_	FICI	Gain	MIC_c1_/MIC_o_	MIC_c2_	FICI	Gain	MIC_c1_/MIC_o_	MIC_c2_	FICI	Gain
Tbc/MC	0.563		0.5^a^		–	–	–	–	–	–	–	–	1.125		0.26^a^		–	–	–	–
Ciprofloxacin	72	18		**4**	–	–	–	–	–	–	–	–	288	2.25		**128**	–	–	–	–
Tb/Ro	0.0702		0.5^a^		–	–	–	–	4.5		0.5^a^		–	–	–	–	–	–	–	–
Ciprofloxacin	72	18		**4**	–	–	–	–	288	72		**4**	–	–	–	–	–	–	–	–
Tb/Ts	0.0702		0.5^a^		–	–	–	–	–	–	–	–	–	–	–	–	–	–	–	–
Ciprofloxacin	72	18		**4**	–	–	–	–	–	–	–	–	–	–	–	–	–	–	–	–
Tb/Tp	0.0011				0.0022				–	–	–	–	–	–	–	–	0.0087			
Ciprofloxacin	72	72	1.25^b^		4.5	0.0351	0.26^a^	**128**	–	–	–	–	–	–	–	–	72	1.13	0.27^a^	**64**
Tb/Tpo	0.281		0.5^a^		–	–	–	–	–	–	–	–	–	–	–	–	–	–	–	–
Ciprofloxacin	72	18		**4**	–	–	–	–	–	–	–	–	–	–	–	–	–	–	–	–

MIC_o_, MIC of the individual EOs in µg/mL; MIC_c1_, MIC of EOs in combination in mg/mL; MIC_c2_, MIC of EOs combinations in association with antibiotics in µg/mL; FICI, Fractional inhibitory concentration index; – not-tested

^a^synergism

^b^indifference

^c^Codes of species as listed in Table 1

## Discussion

The emergence of antibiotic-resistant bacterial pathogens has rendered most available antibiotics ineffective [24]. Alternative strategies are, therefore, needed to counteract drug-resistant bacterial infections. Combination therapies between EOs and conventional drugs to enhance their efficacy appear to be the most effective solution. In this study, the antibacterial effect of Moroccan endemic *Thymus broussonnetii* EO alone, and in combination with EOs obtained from some other medicinal plants namely, *Myrtus communis*., *Artemisia herba alba*., *Thymus pallidus*., *Thymus satureioides*., *Teucrium polium*., and *Rosmarinus officinalis* were evaluated for the first time against some well-known multidrug-resistant bacteria, namely *S. aureus*, *B. subtilis*, *E. coli*, *E. faecalis*, *L. monocytogenes,* and *S. enterica*. The synergistic interactions between the most effective EO combinations (based on *T. broussonnetii* EO) with two conventional antibiotics (streptomycin and ciprofloxacin) were also investigated.

The EOs extracted by steam distillation from aerial parts of the investigated plants yielded variable values, which are consistent with those reported in several previous studies [25–30]. The chemical analyses showed different qualitative and quantitative EO profiles. 1,8-cineole was found to be the major constituent characterizing *M. communis* and *R. officinalis* EOs, which is in agreement with results previously reported by Farah et al. [28] and Pesavento et al. [31]. Thymol was the major compound in the *T. pallidus* EO, while borneol and carvacrol were the most abundant constituents in *T. satureioides* and *T. broussonnetii* EOs, respectively, which are in accordance with findings of Mohamed et al. [30], El Asbahani et al. [32], and Tagnaout et al. [33]. The most abundant compound in *A. herba alba* was β-thujone, while the main constituents of *T. polium* EO were germacrene and α-pinene; these findings are in line with those reported by Barroso et al. [26] and Belmekki et al. [34].

*Thymus* species EOs exhibited significant antibacterial activity against all tested bacteria. Interestingly, *T. broussonnetii* EO exhibited the highest antibacterial efficacy, which is mainly attributable to high levels of its bioactive isomeric monoterpenoid constituents, carvacrol and thymol [35]. The antibacterial activity of these phenolic monoterpenes has been attributed to their chemical structure characterized by the presence of the hydroxyl group, and their strong synergistic effect when applied together [36]. It has been well established that these phenolic monoterpenes can exert their antimicrobial effect through several modes of action against different sites at the cellular level including: disruption of bacterial inner membranes making the structures more permeable, inhibition of microbial motility, inhibition of microbial ATPase, and/or inhibition of efflux pump [13, 37, 38]. The presence of other compounds, including p-cymene, borneol, and γ-terpinene may also contribute to the activity of these oils [39]. The strong antibacterial activity observed in *Thymus* EOs is consistent with reports from previous studies [13, 40, 41]. The EOs of *M. communis* and *R. officinalis* were found to be have antimicrobial activity but at levels lower than *Thymus* spp. EO. This moderate activity mainly due to their richness in some monoterpenes (e.g., 1,8-cineole and α-pinene), well known for their moderate antibacterial potency [42]. The results overall showed that the studied EOs were more effective against Gram-positive bacteria than Gram-negative ones, which is consistent with many previous reports, likely related to their different bacterial cell coating structures [13, 43, 44]. Among the Gram-positive bacteria tested, *S. aureus* was the most sensitive strain to all EOs.

With regard to synergistic interactions between *T. broussonnetii* EO and the other plant EOs, the combination of *T. broussonnetii* and *T. pallidus* showed the highest synergistic antibacterial effects against *S. aureus*, *E. coli,* and *S. enterica*. This result may be attributable to the positive interaction between their major constituents, in particular the phenolic monoterpenes thymol and carvacrol, and with their precursors γ-terpinene and p-cymene [38, 45]. The synergistic antibacterial action between carvacrol and thymol has been ascribed to their capacity to act at different microbial targets [46]. The positive interaction between carvacrol and/or thymol and the main precursors in their biogenetic pathway, p-cymene, and γ-terpinene has been demonstrated in previous reports [47]. Indeed, it has been reported that the hydrophobic nature of p-cymene and γ-terpinene, leads to a swelling of the cytoplasmic membrane that can permit carvacrol and/or thymol to be more easily transported into the bacterial cells [48]. Synergistic interaction associated the binary EO combinations of *T. broussonnetii* and *M. communis*, *R. officinalis*, *T. pallidus*, *T. satureioides,* or *T. polium* were demonstrated against *S. aureus*. In addition, the combinations of *T. broussonnetii* and *R. officinalis*, and *T. broussonnetii* and *M. communis* EOs showed synergism against *E. faecalis* and *L. monocytogenes*, respectively. This can be explained by the chemical profiles of these EOs, which are characterized by the abundance of carvacrol and 1,8-cineole. The synergistic interaction between carvacrol and 1,8-cineole has been previously reported detected against several microorganisms [49, 50], and it has been demonstrated that the combination of carvacrol and 1,8-cineole can disrupt cell membrane permeability of bacterial cells, resulting in the outflow of intracellular functional substances, disturbing normal cell functions, and destroying the cell structure, leading to inactivation of the cells [51]. The synergistic effects of combined *T. broussonnetii* and *T. polium* EOs against *S. aureus* may be the result of a possible positive interaction between carvacrol and α-pinene and/or germacrene.

The interaction of EOs with antibiotics is one of the promising ways to overcome the counter resistance in pathogenic bacteria. A strong synergistic effect was demonstrated between *T. broussonnetii* and *T. pallidus* EO mixture with the two tested antibiotics. This results is likely to be a result of the previously reported positive interactions between the phenolic monoterpenes carvacrol and thymol with the antibiotics, [52, 53]. There was also high synergistic activity from the combination of *T. broussonnetii* and *M. communis* EO mixture with each of the antibiotics.

## Conclusions

Our results demonstrate that EO obtained from *T. broussonnetii* has substantial antibacterial activity, even higher than the two tested antibiotics against some bacterial strains. Combining the EO of this Moroccan endemic *Thymus* species with some other selected plant EOs showed strong synergistic activity against some multidrug-resistant bacteria, with superior synergism when *T. broussonnetii* EO was combined with *T. pallidus* EOs. Furthermore, this effective EO mixture synergistically enhanced the antimicrobial activity of the two antibiotics streptomycin and ciprofloxacin against a range of bacterial strains. This interesting EO mixture is, therefore, suggested as a potential natural adjuvant to potentiate these two conventional antimicrobials, and possibly others. Other effective EO combinations containing *T. broussonnetii* EO were also recorded. Most of these efficacious EO mixtures enhanced the antimicrobial activity of the two tested antibiotics, but to varying degrees and also differed with different bacterial strains. Overall, these results represent the basis for further in vivo investigations, which could ultimately lead to development of new antimicrobial agents based on *T. broussonnetii* EO, and therefore, contribute to enhancing the efficacy of antibiotics in controlling multidrug-resistant pathogenic bacteria. However, additional investigations are needed to determine the compounds responsible for the antibacterial activity in complex mixtures and to elucidate the likely modes of action responsible for their synergistic effect when combined with antibiotics.

## Data Availability

The manuscript includes all the research data used in this study.

## Abbreviations

EOs: Essential oils
AMR: Antimicrobial resistance
EU: European Union
GC/FID: Gas chromatography and flame ionization detector (FID)
GC/MS: Gas chromatography coupled with mass spectrometry
MHA: Mueller Hinton Agar
MHB: Mueller Hinton Broth
DMSO: Dimethyl sulfoxide
CLSI: Clinical and Laboratory Standards Institute
FICI: Fractional Inhibitory Index
IZ: Inhibition Zone Diameters
MIC: Minimum inhibitory Concentration
MBC: Minimum Bactericidal Concentrations
